# Root Traits Enhancing Rice Grain Yield under Alternate Wetting and Drying Condition

**DOI:** 10.3389/fpls.2017.01879

**Published:** 2017-10-31

**Authors:** Nitika Sandhu, Sushil R. Subedi, Ram B. Yadaw, Bedanand Chaudhary, Hari Prasai, Khandakar Iftekharuddaula, Tho Thanak, Vathany Thun, Khushi R. Battan, Mangat Ram, Challa Venkateshwarlu, Vitaliano Lopena, Paquito Pablico, Paul C. Maturan, Ma. Teresa Sta. Cruz, K. Anitha Raman, Bertrand Collard, Arvind Kumar

**Affiliations:** ^1^International Rice Research Institute, Los Baños, Philippines; ^2^National Rice Research Program, Hardinath, Nepal; ^3^Regional Agriculture Research Station, Tarahara, Nepal; ^4^Bangladesh Rice Research Institute, Gazipur, Bangladesh; ^5^Cambodian Agricultural Research and Development Institute, Phnom Penh, Cambodia; ^6^Rice Research Station, Kaul, India; ^7^South Asia Breeding Hub, International Rice Research Institute, International Crops Research Institute for the Semi-Arid Tropics (ICRISAT), Hyderabad, India

**Keywords:** alternate wetting and drying, rice, root, water, yield

## Abstract

Reducing water requirements and lowering environmental footprints require attention to minimize risks to food security. The present study was conducted with the aim to identify appropriate root traits enhancing rice grain yield under alternate wetting and drying conditions (AWD) and identify stable, high-yielding genotypes better suited to the AWD across variable ecosystems. Advanced breeding lines, popular rice varieties and drought-tolerant lines were evaluated in a series of 23 experiments conducted in the Philippines, India, Bangladesh, Nepal and Cambodia in 2015 and 2016. A large variation in grain yield under AWD conditions enabled the selection of high-yielding and stable genotypes across locations, seasons and years. Water savings of 5.7–23.4% were achieved without significant yield penalty across different ecosystems. The mean grain yield of genotypes across locations ranged from 3.5 to 5.6 t/ha and the mean environment grain yields ranged from 3.7 (Cambodia) to 6.6 (India) t/ha. The best-fitting Finlay-Wilkinson regression model identified eight stable genotypes with mean grain yield of more than 5.0 t/ha across locations. Multidimensional preference analysis represented the strong association of root traits (nodal root number, root dry weight at 22 and 30 days after transplanting) with grain yield. The genotype IR14L253 outperformed in terms of root traits and high mean grain yield across seasons and six locations. The 1.0 t/ha yield advantage of IR14L253 over the popular cultivar IR64 under AWD shall encourage farmers to cultivate IR14L253 and also adopt AWD. The results suggest an important role of root architectural traits in term of more number of nodal roots and root dry weight at 10–20 cm depth on 22–30 days after transplanting (DAT) in providing yield stability and preventing yield reduction under AWD compared to continuous flooded conditions. Genotypes possessing increased number of nodal roots provided higher yield over IR64 as well as no yield reduction under AWD compared to flooded irrigation. The identification of appropriate root architecture traits at specific depth and specific growth stage shall help breeding programs develop better rice varieties for AWD conditions.

## Introduction

Increased demand of water for household, industry, agriculture and the changing climatic conditions in term of decreasing monsoon rainfall in South Asia and South East-Asia has made water a more valuable commodity than ever before. “More rice with less water” is vital for water-food security and agriculture sustainability (Tuong et al., 2005). Water shortage has a critical impact on the world's food self-sufficiency and security (FAO, 2016). Farmers in Asia depend mostly on monsoon rains. With the existing climatic vulnerability, almost half the planet's population will be living in areas of high water shortage by 2030 (UNCCD, 2014). In the dry season or in an environment with an evaporation rate higher than the precipitation rate, approximately 700–1,500 mm of water, depending on soil characteristics, is required to produce 1 kg of rice when using traditional practices of rice cultivation (Bhuiyan, 1992). The actual water requirement for rice crop cultivation is much lower than the amount of water traditionally used (Tuong, 1999; Li et al., 2006). This calls attention to the necessity of developing climate-smart, water-saving irrigation (WSI) technologies that reduce water use, carbon sequestration and greenhouse gas (GHGs) emissions (IRRI, 2009; Yang et al., 2017); and the identification of suitable traits leading resilience (adaptation) and stable high yielding genotypes with high water use efficiency and sustainable productivity.

The alternate wetting and drying (AWD) system of irrigation is one of the most common water-saving techniques in practice (Bouman and Tuong, 2001; Belder et al., 2004; Moya et al., 2004). It deals with the problem of water shortage in irrigated rice cultivation and has the potential to contribute to more sustainable and effective water and energy use. AWD is the practice of allowing the field to periodically dry and rewet throughout the growing season instead of keeping paddies in a permanently flooded state. Depending on the soil's type, texture and characteristics; the climate and the crop's development stage, the AWD cycle (flooded-nonflooded) can vary from 1 to 10 days or even more. AWD is a simple, farmer-friendly practice that uses a single device (a water pipe) designed to observe the water level in a rice field for deciding when to irrigate. Compared to flooded/aerobic cultivation, field leveling is critical when using AWD. AWD can contribute to agricultural sustainability by reducing water use in irrigated rice by 15–30% (Zhang et al., 2009), increasing rice yield by approximately 10% relative to continuous flooding (Yang et al., 2007; Zhang et al., 2009), boosting nutritional status (Wissuwa et al., 2008), and decreasing toxic elements such as cadmium (Cd) (Yang et al., 2009) and arsenic (As) (Xu et al., 2008; Yang et al., 2017). The Iron (Fe) toxicity because of high availability of Fe under continuously flooded soils as practiced in some of the coastal areas in Asia and Africa can also be reduced using AWD (Cherif et al., 2009).

In some studies, the use of AWD has been shown to provide similar rice yields to those of continuously flooded systems (Yao et al., 2012) or slightly lower yields (Yadav et al., 2012). The amount of water saved involving AWD would in itself be adequate to rationalize any inconsequential yield loss when using this practice. AWD improves or modifies root/shoot growth and activity (Sarkar, 2001; Yang et al., 2009; Yang and Zhang, 2010) and water use efficiency (Tabbal et al., 2002); facilitates further access to water and nutrients at 0–30 cm soil depth; improves the proportion of productive tillers, alters leaf angle and plant hormone signaling (Davies et al., 2011); and enhances the grain filling rate (Zhang et al., 2010, 2012). AWD technology has a high potential for breeders to develop water-saving and yield-stable rice genotypes by further modifying the root system architecture of presently cultivated rice genotypes. The adaptability of the root system relative to the timing and intensity of fluctuations in soil metric and redox potential will affect the impact of the AWD treatment on nutrient access, whole root system water potential, signaling, resource allocation and partitioning between root and shoot. The critical concerns in the adoption of AWD are the unavailability of adequate knowledge on traits suitable to obtain higher yield under AWD system and the appropriate modification of the root systems of presently available flooded irrigated system adapted genotypes. So far, no rice variety with appropriate root architecture has been developed that produced similar yield across rice growing conditions under AWD. Till date, no study has reported the effect on grain yield under AWD by differences in the root traits at different soil depth and at specific plant growth stage.

The present study was conducted with the aim to identify appropriate root traits enhancing rice grain yield under AWD and identify stable, high-yielding genotypes better suited to the AWD across variable ecosystems. We hypothesized that the rice genotypes with comparatively better root system in the 15–20 cm root zone shall have stable and high grain yield under AWD compared to genotypes with lesser nodal roots and lower root dry weight.

## Materials and methods

### Plant material and locations

The plant material was comprised of advanced breeding lines from irrigated and rainfed breeding programs of International Rice Research Institute (IRRI). The popular rice varieties grown in different countries (PSBRc 52, Matang 1, Matang 9, PSBRc 28, PSBRc 82, BR28, BR29, Janaki, Khao Dawk Mali 105, WS 91, Abhaya, Vasistha, Mahsuri, Teqing, MRQ74, Samba Mahsuri, IR07F287, Fedearroz 50/NSICRc 158, IRRI 143, IRRI 149, IRRI 150, IRRI 168, Apo, Thadokkham 1, NSICRc 138, PSBRc 10, NSICRc 110, MTU1010, and IR64) and drought tolerant breeding lines (IR 77298-14-1-2-10, IR 77298-5-6-18, IR 84984-83-15-18-B-B, IR 81896-B-B-236, IR 78875-176-B-2) were used to develop the advanced breeding lines.

**Figure 1:**
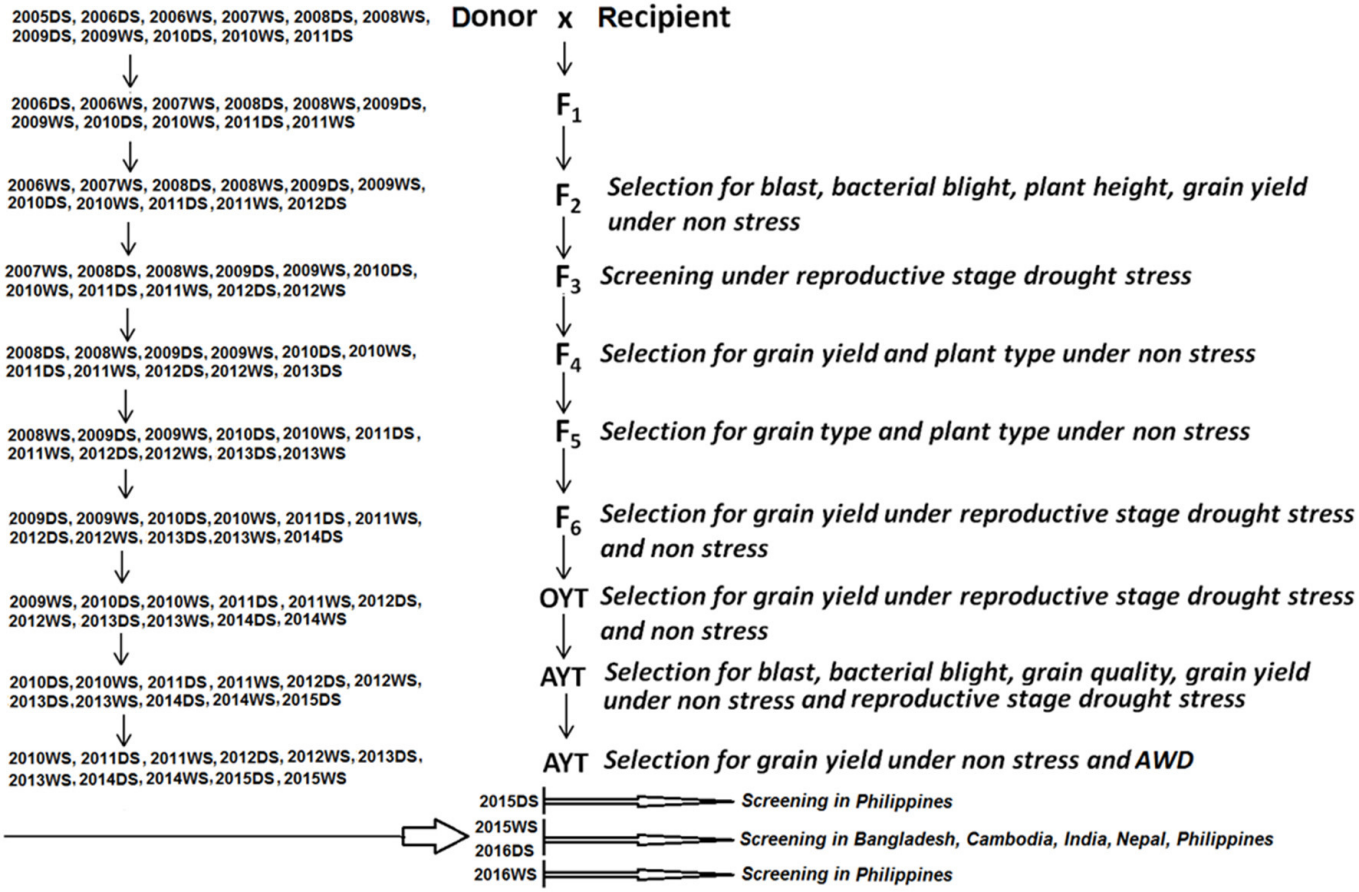
Scheme for the development of advanced breeding lines and selection strategy in generation using a modified conventional breeding approach.

The hybridization program to develop appropriate breeding lines involving the above parents was initiated in 2005DS (Dry Season) with the inclusion of new crosses each successive season. The crossing, selection and advancement scheme is shown in Figure 1. The screening material for AWD experiments includes advanced breeding sister lines from 2005DS (two families), 2006DS (one family), 2006WS (Wet season) (two families), 2007WS (nine families), 2008WS (two families), 2008DS (two families), 2009DS (four families), 2009WS (four families), 2010DS (ten families), 2010WS (16 families), and 2011DS (15 families) (Supplementary Table 1).

To identify promising yield stable genotypes, a series of 23 experiments were conducted under the AWD system of rice cultivation at the IRRI, Philippines during 2015DS, 2015WS, 2016DS, and 2016WS); IRRI-South Asia Breeding Hub, ICRISAT, Hyderabad, India; Bangladesh Rice Research Institute, BRRI, Gazipur, Bangladesh; National Rice Research Program NRRP, Hardinath, Nepal; Regional Agriculture Research Station, RARS, Tarahara Nepal; and Cambodian Agricultural Research and Development Institute, CARDI, Phnom Penh, Cambodia in 2015WS and 2016DS; at Rice Research Station, Kaul, Haryana (India) in 2016WS. Table 1 shows a detailed description of the number of lines screened for each season under different irrigation and management practices. The lines were selected based on no yield reduction under AWD compared to continuous flooding irrigation system for the successive seasons screening at IRRI. At IRRI, in the 2016DS, four genotypes were selected based on yield stability across treatments and seasons and were screened for root and agronomic traits in 2016WS together with lowland adapted check (IR64).

**Table 1:** Details of the experiments conducted in 2015-16 across different ecosystems.

**Experimental site**	**Season**	**Trt**	**No of lines**	**Seeding/transplanting date**	**Experimental details**	**Fertilizer rate**
IRRI, Philippines	2015DS	NS	70	18 December 2015/8 January 2015	(10 × 7) AL with two replications, 8 rows of 5 m each with 0.2 m row spacing	120:30:30 N:P_2_O_5_:K_2_O
		AWD	70	5 January 2015/27 January 2015		120:30:30 N:P_2_O_5_:K_2_O
		AWD	130	7 January 2015/2 February 2015	(10 × 13) AL with two replications, 10 rows of 5 m each with 0.25 m row spacing	120:30:30 N:P_2_O_5_:K_2_O
	2015WS	NS	88	15 June 2015/7 July 2015	(8 × 10) AL with two replications, 8 rows of 5 m each with 0.2 m row spacing	90:25:25 N:P_2_O_5_:K_2_O
		AWD	88	15 June 2015/7 July 2015		90:25:25 N:P_2_O_5_:K_2_O
		AWD	64	19 June 2015/12 July 2015	(8 × 8) AL with two replications, 10 rows of 5 m each with 0.25 m row spacing	90:25:25 N:P_2_O_5_:K_2_O
	2016DS	AWD	28	18 December 2016/8 January 2016	RCBD with 4 rows of 5 m each with 0.2 m row spacing	120:30:30 N:P_2_O_5_:K_2_O
	2016WS	AWD	11	13 June 2016/7 July 2016	RCBD with 4 rows of 5 m each with 0.2 m row spacing	90:25:25 N:P_2_O_5_:K_2_O
IRRI-SA Hub, Hyderabad, India	2015WS	AWD	60	8 June 2015/30 June 2015	Augmented design with 4 rows of 4 m each with 0.2 m row spacing	120:60:40 N:P_2_O_5_:K_2_O
	2016 DS	AWD	30	23 December 2015/29 January 2016		
	2016 DS	AWD	50	23 December 2015/29 January 2016	(10 × 3 AL) with 4 rows of 3 m each with 0.2 m row spacing	
Rice Research Station, Kaul, Haryana, India	2016WS	NS	6	05 July 2016/22 July 2016	RCBD with 4 rows of 4 m each with 0.2 m row spacing	120:60:25 N:P_2_O_5_: ZnSO_4_
	2016WS	AWD	6	05 July 2016/22 July 2016	RCBD with 4 rows of 4 m each with 0.2 m with row spacing	120:60:25 N:P_2_O_5_: ZnSO_4_
NRRP, Hardinath, Nepal	2015WS	AWD	195	9 June 2015/2 July 2015	(39 × 5) AL with two replications with two replications, 5 rows of 5 m each with 0.2 m row spacing	80:40:30 N:P_2_O_5_:K_2_O
	2016 DS	AWD	38	19 Feb 2016/10 March 2016	RCBD with two replications with two replications, 5 rows of 5 m each with 0.2 m row spacing	100:40:30 N:P_2_O_5_:K_2_O
RARS, Tarahara, Nepal	2015WS	AWD	195	16 July 2015/7 August 2015	(39 × 5) AL with two replications, 5 rows of 5 m each with 0.2 m row spacing	80:40:30 N:P_2_O_5_:K_2_O
	2016 DS	AWD	36	19 Feb 2016/8 March 2016	(12 × 3) AL with two replications, 5 rows of 5 m each with 0.2 m row spacing	100:40:30 N:P_2_O_5_:K_2_O
BRRI, Gazipur, Bangladesh	2015WS	AWD	60	24 July 2015/22 August, 2015	(12 × 5) AL with 6 rows of 4.5 m each with 0.25 m row spacing	210:65:85:56:5 Urea:TSP (Triple Superphosphate):MOP (murate of potash):Gypsum:ZnSO_4_
	2016 DS	AWD	42	10 December 2015/23 January, 2016	Augmented design with 4 rows of 5.4 m each with 0.25 m row spacing	300:90:125:55:6 Urea:TSP (Triple superphosphate):MOP (murate of potash):Gypsum:ZnSO_4_
CARDI, Phnom Penh, Cambodia	2015WS	NS	60	30 July 2015/20 August 2015	Augmented design with 6 rows of 5 m each with 0.2 m with row spacing	60:30:30 Urea:DAP:KCl
	2016DS	AWD	60	16 Feb 2016/9 March 2016		
	2016DS	AWD	39	16 Feb 2016/9 March 2016	RCBD with 6 rows of 5 m each with 0.2 m with row spacing	
	2016DS	AWD	39	16 Feb 2016/9 March 2016		

*DS, dry season; WS, wet season; NS, control continuous flooding; AWD, alternate wetting and drying; AL, alpha lattice; RCBD, randomized complete block design; m, meter*.

### Agronomic management and AWD system implementation

Along with AWD experiment, a concurrent controlled experiment was undertaken in which approximately 5 cm of standing water was maintained in the field after transplanting till 10 days before harvesting (Table 1). The detailed description of experiments and management practices is given in Table 1.

The depth of water in the AWD field was measured by installing water pipes (35 cm high × 20 cm diameter, with 5 mm diameter holes and 2 cm spacing from hole to hole) (Supplementary Figure 1) in zig-zag pattern across field. The water level was maintained at 5 cm until 15 days after transplanting (DAT). Then AWD cycle was initiated. When the water level dropped to 15 cm below the soil surface (Supplementary Figures 1, 2), the field was irrigated to the depth of 5 cm. A week before to a week after the peak of flowering, water was maintained to 5 cm depth to avoid water stress. AWD cycle was re-started after flowering and during grain filling. The number of irrigation events was noted throughout the experiment. Rainfall data were also taken into consideration in calculating the water saving achieved. The amount of water used and water saved (in percentage) were calculated as:

$$\begin{array}{l} Amount\;of\;water\;used:\;(Total\;number\;of\;irrigation\;\times \;Total\;area\;\times \;Depth\;of\;ponding\;water)\;+\;(Total\;rainfall)\\ \;\%\;water\;saving:\;\frac{Water\;used\;in\;continuous\;irrigation\;(flooded)\;-Water\;used\;in\;AWD}{Water\;used\;in\;continuous\;irrigation\;(flooded)}\times \;100 \end{array}$$

### Data collection

#### Agronomic traits

The number of days to flowering was recorded when 50% of the plants in the plot exerted their panicles. At maturity, plant height was measured from the base of the plant to the tip of the highest panicle from three random plants per plot. The harvested grains were threshed and oven dried for 3 days (at 50°C). The grain weight data was normalized to a moisture content of 14% to estimate grain yield (t/ha). Similar measurements were done at the locations in India, Bangladesh, Nepal and Cambodia.

#### Seedling and root traits

**Figure 2:**
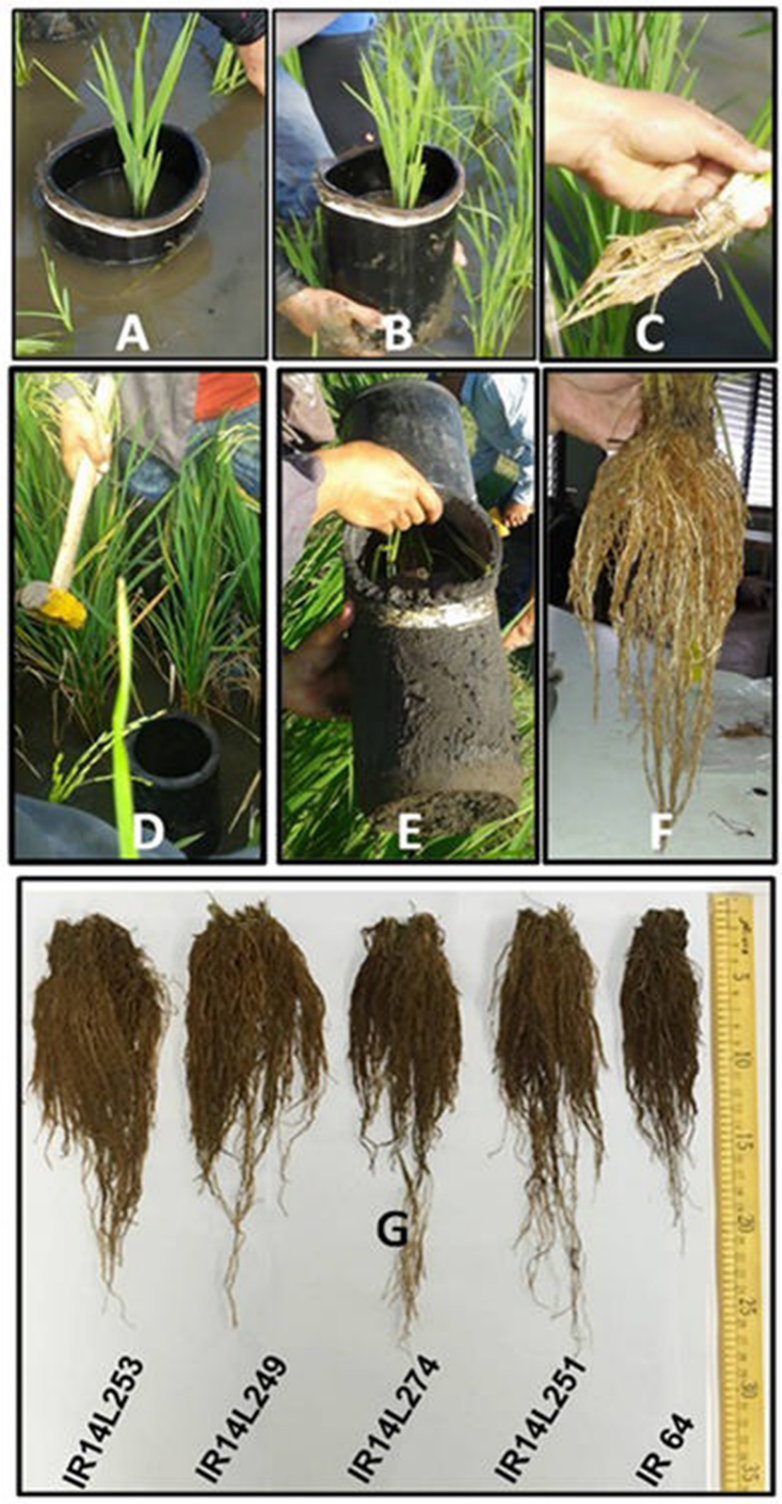
**(A–C)** Root sampling at 15 days after transplanting in field. **(D–F)** Root sampling at 50% flowering. **(G)** Comparison of root system of selected lines and check (IR64) at flowering stage at IRRI, Philippines.

During the 2016WS, three seedlings per plot were sampled by digging a hole in the soil using whole plant core sampler at 15, 22, 30 DAT and at 50% days to flowering (Figure 2). The roots were gently washed over a sieve. The number of nodal roots per plant (NR) was counted manually. The root length (RL) was measured with centimeter scale. The roots and shoots were then separated and shoots were dried in an oven at 60°C for 3 days to record root dry weight (RDW) and shoot dry weight (SDW). The relative growth rate (RGR) was calculated in term of shoot dry weight as follows:

$$RGR=\left[\frac{ln\;(shoot\;dry\;weight\;at\;sampling2)-ln\;(shoot\;dry\;weight\;at\;sampling1)}{\left(date\;of\;sampling\;2\;-\;date\;of\;sampling\;1\right)}\right]$$

### Data analysis

#### Trial-wise analysis

Analysis of variance (ANOVA) for seedling, root and agronomic traits from each trial (site × year × season) was performed using the SAS PROC MIXED (Littell et al., 2006) taking replications and blocks within replications as random effects and lines as fixed effects. Broad-sense heritability (H) was estimated as:

$$H=\frac{\sigma^{2}g}{\sigma^{2}g+\frac{\sigma^{2}e}{r}}$$

where $\sigma_{g}^{2}$ is the genotypic variance, $\sigma_{e}^{2}$ the error variance, and *r* the number of replications.

#### Across trial yield stability analysis

The genotypes that were tested in four or more trials under AWD were included in the yield stability analysis. Four conventional stability models with homogenous and heterogeneous error variances were fitted to the genotype x trial table of means within the mixed model framework Piepho (1999) in which the trials were treated as random and genotypes as fixed. These included (i) Shukla's stability variance model (ii) Finlay-Wilkinson model (iii) Eberhart-Russell model, and (iv) AMMI model. The best-fitting model was selected based on the lowest Akaike Information Criterion (AIC) value.

The mean grain yield across trials for the selected lines and their characterized root and shoot trait observations were displayed on a MDPREF (multidimensional preference) biplot using SAS PROC PRINQUAL procedure (Caroll, 1972; Kuhfeld, 1992; Linting et al., 2007). MDPREF identifies the variability that is most salient to the preference patterns of the traits toward the genotypes and extracts this as the first principal component (Singh et al., 2001, 2017). The second principal component represents the direction that is most salient to the preferences that are orthogonal to the first principal component).

## Results

### Phenotypic screening across seasons and locations

At IRRI, under non stress (continuous flooding) the mean days to 50% flowering ranged from 85 to 88 days, mean plant height ranged from 95 to 120 cm and mean grain yield ranged from 5.3 to 7.4 t/ha. Under AWD conditions the mean days to 50% flowering ranged from 79 to 89 days, mean plant height from 95 to 121 cm while mean grain yield from 4.8 to 7.5 t/ha at IRRI. The trial heritability's for grain yield varied from 0.52 to 0.92 at IRRI (Table 2). At IRRI, 12% reduction in grain yield was observed under AWD compared to continuous flooding (the control conditions) in 2015DS while in 2015WS, the yield under AWD was 9% higher than continuous flooding control. The grain yield was 2% and 6–8% higher under AWD conditions compared to continuous flooding control conditions in India and Cambodia, respectively. In India the mean days to 50% flowering ranged from 75 to 111 days, mean plant height from 81 to 117 cm and mean grain yield from 3.5 to 6.7 t/ha, with heritability for grain yield 0.41 to 0.85 under AWD. In Nepal, the variability in genotypes under AWD ranged from 88 to 113 days for days to 50% flowering, 85 to 95 cm in term of mean plant height and 2.9 to 5.4 t/ha for mean grain yield (Table 2). At Gazipur, Bangladesh the mean grain yield under AWD varied from 4.0 to 4.3 t/ha with estimated heritability ranging from moderate to high (0.61 to 0.98). At Phnom Penh, Cambodia the mean grain yield variability ranged from 3.4 to 3.8 t/ha and 3.6 to 4.1 t/ha under continuous flooding and AWD, respectively (Table 2).

**Table 2:** Descriptive agronomic traits statistics under different treatments and across ecosystems with experimental details.

**Exp**	**Location**	**Year/season**	**Trt**	**Experimental design**	**No. of rep**	**No. of entries**	**Trial mean**			**Trial H**
							**DTF**	**PHT**	**GY**	**GY**
1	IRRI, Philippines	2015DS	NS	(10 × 7) AL	2	70	85	95	7.4	0.52
2		2015DS	AWD	(10 × 7) AL	3	70	86	95	6.5	0.75
3		2015DS	AWD	(10 × 13) AL	2	130	88	107	7.2	0.79
4		2015WS	NS	(8 × 11) AL	2	88	88	120	5.3	0.82
5		2015WS	AWD	(8 × 11) AL	2	88	84	118	5.7	0.71
6		2015WS	AWD	(8 × 8) AL	2	64	89	121	4.8	0.81
7		2016DS	AWD	RCBD	2	28	79	105	7.5	0.64
8		2016WS	AWD	RCBD	2	11	82	117	5.1	0.92
9	Hyderabad, India	2015WS	AWD	(3 × 60) augmented RCBD	–	60	75	100	6.7	0.85
10		2016DS	AWD	(3 × 10) AL	2	30	97	87	5.8	0.41
11		2016DS	AWD	(3 × 50) augmented RCBD	–	50	111	81	3.5	0.42
12	RRS, Kaul, India	2016WS	NS	RCBD	2	6	78	114	5.8	0.84
13		2016WS	AWD	RCBD	2	6	79	117	5.7	0.87
14	Hardinath, Nepal	2015WS	AWD	(39 × 5) AL	2	195	91	99	3.5	0.53
15		2016DS	AWD	RCBD	2	38	97	92	5.4	0.96
16	RARS, Tarahara	2015WS	AWD	(39 × 5) AL	2	195	113	85	2.9	0.65
17		2016DS	AWD	(12 × 3) AL	2	36	88	92	4.0	0.74
18	Gazipur, Bangladesh	2015WS	AWD	(12 × 5) AL	2	60	99	98	4.3	0.98
19		2016DS	AWD	(3 × 42) augmented RCBD	–	42	111	95	4.0	0.61
20	Phnom Penh, Cambodia	2016DS	NS	(2 × 60) augmented RCBD	–	60	80	106	3.4	0.94
21		2016DS	AWD	(2 × 60) augmented RCBD	–	60	80	106	3.6	0.98
22		2016DS	NS	RCBD	2	39	78	89	3.8	0.79
23		2016DS	AWD	RCBD	2	39	106	110	4.1	0.71

*DS, dry season; WS, wet season; NS, control continuous flooding; AWD, alternate wetting and drying; AL, alpha lattice; RCBD, randomized complete block design; rep, replications; DTF, days to 50% flowering (days); PHT, plant height (cm); GY, grain yield (t/ha); H, heritability*.

### Potential economic investment

Total water input across locations ranged from 782 to 1,705.4 m^3^. Total water input was higher during the wet season compared to the dry season. The total amount of water consumed in control (continuous flooding) and AWD treatment with total rainfall across seasons, years and locations is shown in Table 3. Water saving of 18.9 to 22.5, 19.9, 15.4, 23.4, 14.0%, and 5.7 to 7.3% was observed at IRRI, India, Nepal, Bangladesh and Cambodia locations, respectively (Table 3).

**Table 3:** Economic water saving under AWD compared to continuous flooding (control) across different ecosystems.

**Location**	**Seasons/year**	**Water regimes**	**No. of irrigations**	**Irrigation water (m^3^)**	**Rainfall (m^3^)**	**Total water (m^3^)**	**% irrigation water saved over NS**
IRRI	2015DS	NS	24	1, 182.7	129.3	1, 312.0	22.5
		AWD	18	887.0	129.3	1, 016.3	
	2015WS	NS	12	743.4	892.2	1, 635.7	18.9
		AWD	7	433.7	892.2	1, 325.9	
India	2016WS	NS	24	1, 345.0	360.4	1, 705.4	19.9
		AWD	18	1, 005.0	360.4	1, 365.4	
Nepal	2015WS	NS	14	430.0	996.8	1, 426.8	15.4
		AWD	6	210.0	996.8	1, 206.8	
	2016 DS	NS	22	901.0	234.0	1, 135.0	23.4
		AWD	16	635.0	234.0	869.0	
Bangladesh	2016 DS	NS	9	602.0	307.0	909.0	14.0
		AWD	7	487.0	307.0	782.0	
Cambodia	2016DS	NS	19	338.4	917.4	1, 255.8	5.7
		AWD	15	267.1	917.4	1, 184.5	
		NS	19	483.6	917.4	1, 401.0	7.3
		AWD	15	381.8	917.4	1, 299.2	

*DS, dry season; WS, wet season; NS, control continuous flooding; AWD, alternate wetting and drying; m^3^, cubic meter*.

### Mean yield and stability across environments (location × year)

The mean grain yield of genotypes across environments ranged from 3.5 (IR14L255) to 5.6 t/ha (IR14L111, IR14L146, IR14L360, and IR11N313) (Supplementary Table 2). The mean yield of locations over years ranged from 3.7 t/ha (Cambodia; lower) to 6.6 t/ha (India; higher). A total of 33 genotypes had shown mean grain yield of more than 5.0 t/ha across locations. The mean grain yield of 42 genotypes across environments varied from 4.0 to 5.0 t/ha. Most of the stable and high mean grain yielding genotypes across different locations and years/seasons was from rainfed lowland breeding program at IRRI (Supplementary Table 2), indicating the adaptability of lowland adapted genotypes across environments under AWD conditions.

Shukla stability variance model (homogeneous error variance) and Finlay-Wilkinson model (heterogeneous error) could meet the convergence criteria with the AIC values of 1,942.9 and 1,224.9, respectively. Therefore Finlay-Wilkinson model with lower AIC value was considered best suited to the data and the best performers for stability were identified. Eighty two genotypes tested in three or more environments were used to evaluate yield stability of the genotypes across environments. Finlay-Wilkinson model (Finlay and Wilkinson, 1963) with heterogeneous variances for errors was the best fitting stability model. Genotypes IR14L249, IR14L157, IR14L135, IR11N313, IR14L253, IR11A282, IR14L273, IR14L158, IR12N135, and IR14L545 were good performers in terms of stability with regression coefficient approximating unity. Of these IR11N313, IR14L253, IR14L249, IR14L158, and IR14L157 were high yielding. Out of these, 8 genotypes (IR14L249, IRRI 123, IR14L273, IR14L251, IR14L253, IR11N313, IR14L545, IR11A334) had shown yield stability across locations with high mean grain yield performance (more than 5.0 t/ha) (Table 4). It is important to highlight here that the three most stable and high mean grain yielding genotypes (IR14L253, IR14L249, and IR14L273) are the progenies of the crosses involving lowland adapted breeding genotypes-IR71700-247-1-1-2 and IR70181-32-PMI 1-1-5-1, IRRI 163, respectively in their pedigree, indicating that these are potential donors in developing varieties suited to AWD.

**Table 4:** Grain yield stability of selected genotypes across ecosystems based on the best fitting (Finlay-Wilkinson regression model) variance–covariance structure according to the Akaike Information Criterion (AIC).

**Designation**	**Estimate**	**Standard error**	***Z*-value**	**Pr Z**	**Rescaled beta**	**Mean grain yield (t/ha) across locations**
IR11A334	1.6405	0.4426	3.71	0.0002	1.04	5.2
IR11N313	1.5662	0.3280	4.78	<0.0001	0.99	5.6
IR14L249	1.5759	0.3298	4.78	<0.0001	1.00	5.4
IR14L251	1.6804	0.3529	4.78	<0.0001	1.07	5.2
IR14L253	1.5598	0.3263	4.78	<0.0001	0.99	5.5
IR14L273	1.5486	0.3486	4.44	<0.0001	0.98	5.3
IR14L545	1.6394	0.3631	4.52	<0.0001	1.04	5.3
IRRI 123	1.5128	0.3172	4.77	<0.0001	0.96	5.3
IR64	2.1486	0.5971	3.60	0.0003	1.36	4.5

*AIC for the model: 1224.9*.

The mean grain yield performance of genotype IR14L253, IR11N313 was estimated from 6 locations; IR14L273, IR14L249 from 5 locations and IR14L251, IR14L545, IR11A334 from 4 locations. The mean grain yield performance of selected genotypes across ecosystems (Philippines, India, Nepal, Bangladesh and Cambodia) is shown in Table 5. The stable yielding genotypes had shown grain yield advantage of 9.1 to 30.9%, 28.6 to 55.1% and 6.9 to 51.7% over locally adapted check (IR64) in Philippines, India and Nepal, respectively. The percentage increase in grain yield of selected stable genotypes over the locally adapted check BRRI dhan 28 in Bangladesh ranged from 2.0 to 32.7%, and over locally adapted check Chul'sa in Cambodia ranged from 12.8 to 25.6%. The selected genotypes had shown low chalkiness, medium amylose content and high head rice recovery (Table 5), indicating their suitability to be released as a variety for cultivation under AWD system.

**Table 5:** Mean grain yield (t/ha) performance and grain quality parameters of selected genotypes across ecosystems.

**Designation**	**Philippines**	**India**	**Nepal**	**Bangladesh**	**Cambodia**	**Chalkiness**	**Amylose content**	**% Head_Rice**
IR11A334	6.0	6.3	4.3	6.5	–	7.2	22.4	49.0
IR11N313	6.9	7.5	4.4	5.0	4.4	3.5	20.7	49.2
IR14L249	6.7	7.6	3.1	4.9	–	3.3	24.7	59.2
IR14L251	6.9	7.0	3.2	4.6	–	8.7	25.6	52.8
IR14L253	7.2	6.4	4.4	4.4	4.9	10.0	23.0	43.9
IR14L273	6.4	7.6	3.2	5.1	–	2.0	26.5	56.4
IR14L545	7.1	7.6	3.5	4.3	4.4	4.8	24.7	58.6
IRRI 123	6.4	–	3.3	–	–	14.0	22.0	48.9
IR64	5.5	4.9	2.9	–	–	11	21.8	47.6
BRRI dhan 28	–	–	–	4.9	–			
BRRI dhan 56	–	–	–	4.8	–			
Chul'sa	–	–	–	–	3.9			
Overall mean	6.5	6.2	3.9	4.2	3.8			
LSD (5%)	0.95	1.84	1.29	0.59	0.32			
CV (%)	7.74	13.3	15.73	4.82	8.69			

*LSD, least significance difference (5%); CV (%), coefficient of variance; H, heritability*.

Regression coefficient value of a particular genotype across environments provides an estimate about the adaptability of genotype toward different environments and locations and help breeders to select the genotypes. A total of 49 genotypes had shown regression coefficient value (rescaled beta) decreasing below unity (1), out of which 5 genotypes had grain yield less than 4.0 t/ha while 19 genotypes yielded more than 5.0 t/ha across locations, indicating high yield performance of 19 genotypes with higher adaptation to environmental change (rescaled beta >1) (Supplementary Table 2). On other hand 30 genotypes had shown regression coefficient value increasing above unity, out of which 3 genotypes yielded less than 4.5 t/ha and remaining 13 yielded more than 5.0 t/ha across locations, indicating better overall yield performance of 13 genotypes with sensitivity to environmental change (Supplementary Table 2).

### Evaluation of root traits

The genotypes based on their stable yield performance under AWD and continuous flooding control conditions were selected and screened for root traits at IRRI. Grain yield data for 27 genotypes screened at IRRI across seasons (common in 2015DS and 2015WS) under continuous flooding and AWD conditions were subjected to analysis of variance. Four genotypes, which showed non-significant yield differences under AWD and continuous flooding control conditions, were selected for root studies in 2016WS under AWD conditions (Table 6).

**Table 6:** Comparison of grain yield (t/ha) of selected lines under AWD and continuous flooding (control) conditions at IRRI, Philippines.

**Designation**	**2015DS_NS**	**2015DS_AWD**	**Std error of diff**	***F*-value**	**2015WS_NS**	**2015WS_AWD**	**Std error of diff**	***F*-value**	**2016DS_AWD**	**2016WS_AWD**
IR14L249	7.4	6.5^ns^	0.584	2.42	5.9	5.7^ns^	0.335	0.04	8	4.9
IR14L251	7.1	7.0^ns^	0.853	0.03	5.6	5.9^ns^	0.824	0.18	7.8	5.3
IR14L253	7.8	7.8^ns^	0.189	0.25	6.4	5.7^ns^	0.197	7.96	8.3	6
IR14L274	8.2	6.6^ns^	1.6	0.67	5.7	6.0^ns^	0.45	0.58	7.7	4.6
IR64	6.6	5.8^*^	0.099	358.7	5.9	4.8^*^	0.054	430.97	7.4	5.2
Trial mean	7.4	6.5	0.704	2.82	5.3	5.7	0.563	3.4	7.5	5.1
LSD (5%)	1.9	0.91	–	–	1.11	9.9	–	–	0.8	0.87
CV (%)	11.9	7.9	–	–	1.14	9.7	–	–	5.4	6.2

*ns, genotype had no significance difference in yield under NS and AWD conditions across rows*.

* *Significant difference at 0.05*.

*DS, dry season; WS, wet season; NS, control continuous flooding; AWD, alternate wetting and drying; Std error of diff, standard error of difference; LSD, least significance difference (5%); CV (%), coefficient of variance*.

The root traits, nodal root number and root dry weight at 10–20 cm depth on 22–30 DAT and percentage increase in these root traits after initiation of AWD cycle play an important role in maintaining grain yield under AWD in the selected genotypes. The lowland adapted check (IR64) had shown significant grain yield difference with lower yield under AWD conditions compared to continuous flooding control conditions (Table 6). The nodal root number in IR64 was higher compared to the selected genotypes before the initiation of the AWD treatment (15 days after transplanting [DAT]). After the initiation of the treatment, at 22 and 30 DAT, all the four selected genotypes maintained more nodal roots and higher root dry weight compared to lowland adapted check (IR64) (Table 7) between 10 and 20 cm. Percent increase in nodal root number, root length and root dry weight at 22 DAT, 30 DAT and days to 50% flowering was higher in the selected genotypes compared to IR64 (Table 8). Number of nodal root below 20 cm showed significant difference at flowering stage (50% flowering, Table 7). The percent increase in nodal root number and root dry weight from 15 DAT to 22, 15 to 30 DAT, from 15, 22, and 30 DAT to days to 50% flowering were significantly higher in the selected genotypes compared to IR64 (Table 8).

**Table 7:** Comparison of root traits of selected lines under AWD at different sampling times at IRRI.

**Designation**	**1st sampling @ 15 DAT**				**2nd sampling @ 22 DAT**				**3rd sampling @ 30 DAT**				**Sampling at flowering stage**								
	**NR**	**RL**	**RDW**	**SDW**	**NR**	**RL**	**RDW**	**SDW**	**NR**	**RL**	**RDW**	**SDW**	**NR**	**NR > 20 cm**	**RL**	**RDW**	**SDW**	**RGRA**	**RGRB**	**RGRC**	**RGRD**
IR14L249	52	10.8	0.12	0.52	100	12.2	0.425	1.72	127	15.2	0.603	3.96	167	8	24	1.58	19.88	0.17	0.094	0.264	0.256
IR14L251	53	11.2	0.201	0.61	108	13.4	0.512	2.18	135	16.8	0.614	3.68	187	15	29	1.83	17.96	0.183	0.075	0.258	0.226
IR14L253	60	11.1	0.111	0.62	140	12.5	0.459	2.3	163	16	0.588	4.22	198	6	26	1.59	19.84	0.186	0.087	0.273	0.221
IR14L274	62	11.5	0.214	0.79	120	12.5	0.399	1.75	133	15.5	0.544	3.23	179	30	30	1.9	19.57	0.113	0.088	0.201	0.257
IR64	64	10.4	0.198	0.61	84	11.3	0.348	1.41	98	13.6	0.445	3.29	118	5	21	1.1	19.37	0.121	0.121	0.242	0.253
Trial mean	53	11.1	0.186	0.62	101	13	0.357	1.76	124	14.3	8.47	3.44	183	14	27	1.67	20.17	0.147	0.099	0.246	0.253
*F*-value	1.72	0.55	1.59	0.5	4.81^*^	2.42	0.91	2.82	9.04^**^	1.26	12.06^**^	4.64	2.29	5.26^*^	2.87	4	2.88	1.89	2.11	0.53	0.64
CV (%)	14.9	9.9	72.9	34.4	18.2	16.2	33.2	14.9	14.3	8.5	13.5	16.1	18.6	65.8	6.7	20.8	20.3	19.2	18.8	20.4	22.8

*DAT, days after transplanting; NR, number of nodal roots; RL, root length; RDW, root dry weight; SDW, shoot dry weight; RGRA, relative growth rate from 22 DAT to 15 DAT; RGRB, relative growth rate from 30 DAT to 22 DAT; RGRC, relative growth rate from 30 DAT to 15 DAT; RGRD, relative growth rate from 50% flowering to 30DAT*.

* *Significant difference at 0.05*,

** *Significant difference at 0.01*.

**Table 8:** Percent increase in different root traits of selected lines at 22 and 30 DAT and days to 50% flowering under AWD condition at IRRI.

**Designation**	**% increase from 15 DAT to 22 DAT**		**% increase from 22 DAT to 30 DAT**		**% increase from 30 DAT to days to 50% flowering**		**% increase from 15 DAT to 30 DAT**		**% increase from 22 DAT to days to 50% flowering**		**% increase from 15 DAT to days to 50% flowering**	
	**NR**	**RDW**	**NR**	**RDW**	**NR**	**RDW**	**NR**	**RDW**	**NR**	**RDW**	**NR**	**RDW**
IR14L249	92.1	256.1	27.8	42.3	31.1	161.7	145.5	401.8	67.6	270.9	221.9	1,218.9
IR14L251	104.1	157.5	24.3	20.5	39.2	196.6	153.6	213.1	72.9	310.8	252.7	842.6
IR14L253	197.3	257.0	20.5	32.8	47.7	215.2	207.9	271.0	47.5	243.5	317.9	2,581.3
IR14L274	101.2	93.0	10.6	36.3	34.8	247.0	172.8	183.6	49.2	373.6	204.6	836.6
IR64	50.2	61.2	16.9	48.9	20.2	86.4	57.1	112.1	40.5	165.9	89.1	486.7
Trial mean	108.9	164.96	20.01	36.17	34.60	181.38	147.38	236.30	55.52	272.94	217.23	1,193.24
LSD	41.54	111.0	23.50	68.50	10.78	75.08	47.90	108.44	16.76	93.12	93.18	2,596
*F*-value	13.34^**^	5.32^*^	0.6442	0.194	7.11^*^	5.35^*^	10.89^**^	8.05^*^	5.54^*^	5.51^*^	6.44^*^	0.7932
CV (%)	19.07	27.71	18.57	29.94	15.59	20.70	16.25	22.95	15.09	17.06	21.45	34.42

*DAT, days after transplanting; NR, number of nodal roots; RL, root length; RDW, root dry weight; SDW, shoot dry weight; LSD, least significance difference (5%); CV (%), coefficient of variance*.

* *Significant difference at 0.05*,

** *Significant difference at 0.01*.

### Relationships among traits and genotypes

**Figure 3:**
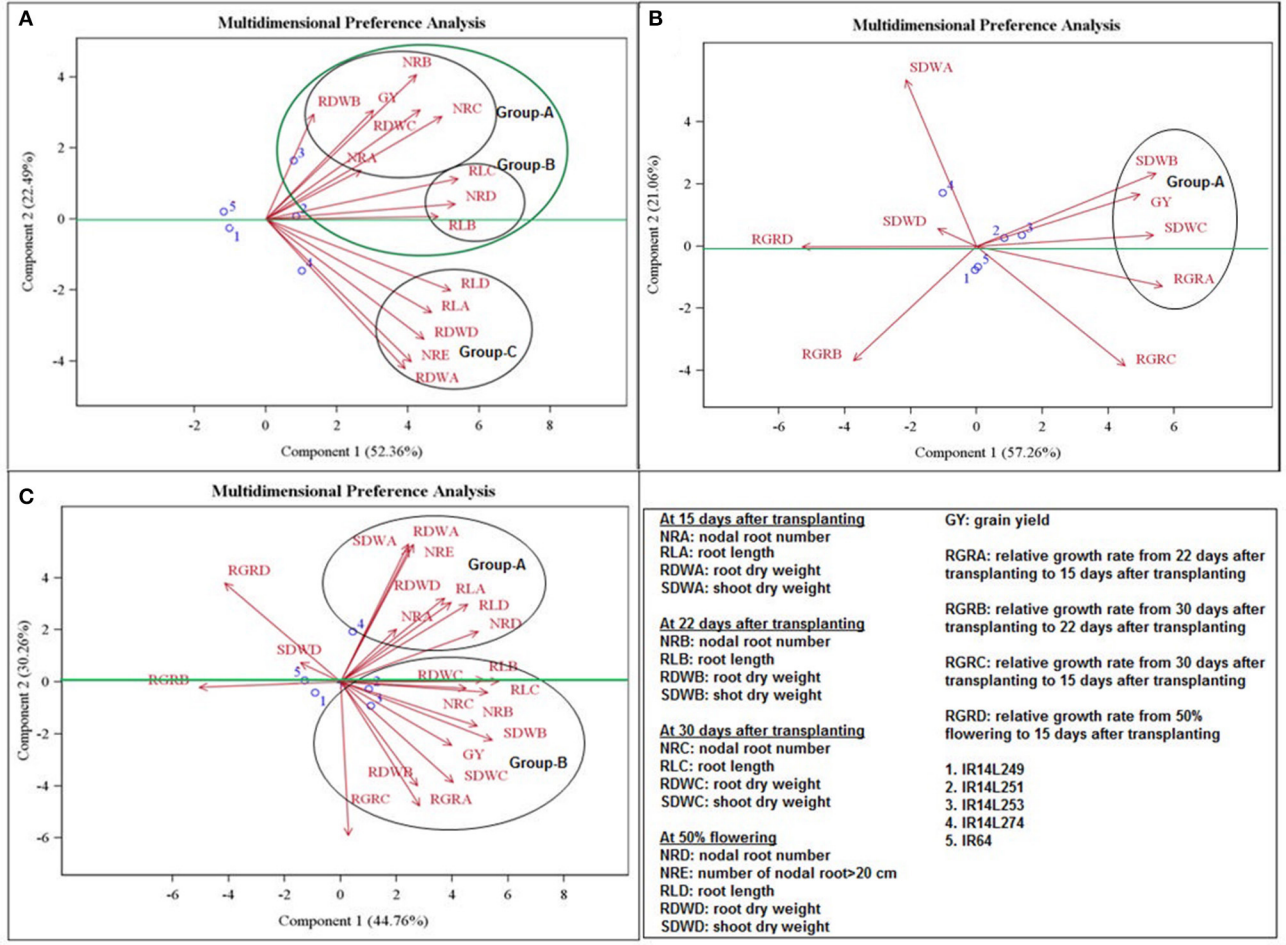
Multidimensional preference analysis biplot of **(A)** root traits with grain yield; **(B)** seedling establishment and shoot traits with grain yield; **(C)** seedling establishment, root and shoot traits with grain yield.

The relationship among the selected genotypes and traits (seedling establishment, root, shoots and grain yield) were examined using multi-dimensional preference analysis (MDPREF) biplots. A trait vector in direction of particular set of genotypes signified the most preferred trait for those genotypes. In each biplot, most of the variability is covered by the first two preference axes and the trait scores are joined to the origin by the trait vectors. The projection of genotype on the trait vector corresponds to the relationship of genotype with the trait. Separate biplots were drawn for root traits vs. grain yield (Figure 3A), seedling establishment (relative growth rate) and shoot dry weight vs. grain yield (Figure 3B) and root, seedling establishment and shoot dry weight vs. grain yield (Figure 3C). The biplots involving different combination of traits showed clearly interpretable dimensions that underlie the data.

The traits at 22 and 30 DAT; root traits such as nodal root number at 15 DAT (NRA), nodal root number at 22 DAT (NRB), nodal root number at 30 DAT (NRC), root dry weight at 22 DAT (RDWB), root dry weight at 30 DAT (RDWC), root length at 22 DAT (RLB), root length at 30 DAT (RLC); shoot traits such as shoot dry weight at 22 DAT (SDWB), shoot dry weight at 30 DAT (SDWC); seedling establishment traits such as relative growth rate from 22 to 15 DAT (RGRA) and relative growth rate from 30 to 15 DAT (RGRC) grouped together (Figure 3). The vectors corresponding to RDW at 22 and 30 DAT; NR at 15, 22, and 30 DAT grouped together with grain yield (GY) (Group-A) were positioned closely on the top right space while the vectors corresponding to RL at 22 and 30 DAT); and nodal root number at days to 50% flowering (NRD) (Group-B) that formed another group were located toward the center on the right (Figure 3A). The vectors corresponding to root length at 15 DAT (RLA) and root length at days to 50% flowering (RLD); root dry weight at 15 DAT (RDWA) and root dry weight at days to 50% flowering (RDWD); and (nodal root number below 20 cm (NRE) (Group-C) tended toward the bottom right space (Figure 3A). Genotypes 3, 2, and 4 were the best performers for the Group-A, B, and C set of traits, respectively. Genotypes 1 and 5 located at the origin were mostly non-responsive (Figure 3A).

For the biplot for shoot and seedling establishment traits with GY, the vector corresponding to SDW at 22DAT, and 30 DAT; RGR from 22 to 15 DAT grouped together with GY (Group-A) were positioned closely toward the center on right whereas other traits were further apart (Figure 3B). Genotypes 2 (IR14L251) and 3 (IR14L253) were the best performers for the Group-A (Figure 3B).

The relationship among traits (seedling establishment, root, shoot and grain yield) and the selected genotypes across the growth period (seedling to maturity) was investigated. The vector corresponding to root traits (NR, RL, and RDW), shoot traits (SDW) at 22 DAT and 30 DAT; seedling establishment traits (RGR from 22 to 15 DAT and from 30 to 15 DAT) grouped together with GY (Group-B). The other measured traits grouped together in Group-A except seedling establishment traits (RGR from 30 to 22 DAT and from days to 50% flowering to 15 DAT); and shoot trait (SDW at days to 50% flowering) (Figure 3C). Genotypes 2 and 3 were the best performers for the Group-B (Figure 3C).

## Discussion

Better crop establishment, high and stable yield, and phenotypic plasticity to adapt across variable growing conditions are the most important reasons for implementing the water-saving investment technologies that will result in a more sustainable agriculture. In the present study, large variability in agronomic traits including grain yield under AWD conditions at different locations, seasons and years resulted from different soil types, characteristics, textures and water holding capacity; soil nutrient dynamics; nutrient cycle; crop fertility maintenance and changing climate (Bouman and Tuong, 2001; Mandal et al., 2009; Price et al., 2013). The economic impact of AWD on saving water (5.7–23.4%) depending on season, without significant yield penalty across different topological ecosystems indicates the potential of technology to address the major challenge of water scarcity in irrigated ecosystems. AWD system of irrigation significantly reduces the water use (by 30%, Bouman et al., 2007, 21–56%, Nalley et al., 2015, 23%, Carrijo et al., 2017; 57% Howell et al., 2015) while maintaining (Howell et al., 2015) or even increasing yields, compared to the traditional continuous flooding system (Mishra et al., 1990; Bouman and Tuong, 2001; Tabbal et al., 2002; Belder et al., 2004; Mandal et al., 2009; Mishra and Salokhe, 2010).

The yield stability and high mean grain yield potential of genotype across variable growing conditions and ecosystems indicate their potential in maintaining proper balance, trade-off and partitioning between root-shoot system. The genotypes with regression coefficient value increasing above 1.0 (Supplementary Table 2) possessed high sensitivity to environmental change, (thus below average stability) and adaptability to favorable environments. Regression coefficient value decreasing below 1.0 (Supplementary Table 2) provides a measure of greater adaptation to environmental change, (thus above average stability) and increasing adaptability to unfavorable environments. This may provide useful information during selection of genotypes with a high mean grain yield and adaptability for specific location. Identification of rice genotypes with high, stable yield under AWD across ecosystems and without any yield penalty under continuous flooding conditions shall promote the adoption and dissemination of water saving AWD technology among farmers. The better grain quality parameters of the selected genotypes indicate the potential of the genotypes to be released as varieties.

The modification of root system architecture of presently cultivated rice genotypes or the identification of stable high yielding genotypes with plasticity in their root systems with change in ecosystems may increase the chances of improving water savings as well as yield under AWD. The adaptive behavior of the root systems of currently available irrigated system genotypes relative to the timing and severity of fluctuating water-nutrient availability is a critical issue in optimizing AWD technology. It is hypothesized that different rice genotypes with comparatively more nodal roots and higher root dry weight in the 15–20 cm root zone are expected to perform better under AWD compared to genotypes with fewer nodal roots within 15–20 cm depth. The fluctuating wetting and drying spells during AWD can improve nutrient cycling processes, microbial dynamics and nutrient mineralization by disrupting the soil aggregates and provoking both physical and biological changes to optimize resource use efficiency to benefit the plant. During AWD treatment, since the water table goes below a depth of 15 cm, 1 or 2 days of delay in irrigation can sometimes reduce the nutrient availability along with the water. In the present study, we measured a number of root traits such nodal root number, nodal root number below 20 cm, root length, root dry weight, root hair length, and root hair density (data not reported) at 15, 22, and 30 DAT and days to 50% flowering. However, only nodal roots and root dry weight showed significant differences. More nodal roots between 0 and 15 cm upto 22 DAT and between 0 and 20 cm thereafter helps breeding lines to have better nutrient uptake, therefore counter the yield reduction under AWD compared to genotypes with lesser nodal root number. The present study underlines importance of presence of higher number of nodal roots and a higher root dry weight between 10 and 20 cm at 22 to 30 DAT in test genotypes producing higher yield under AWD compared to the check IR64. This validates our hypothesis and signifies the suitability of the identified root traits in providing better adaptation under AWD. In our present root study we have also used upland adapted control such as Kali Aus and Aus 276. However, as AWD is transplanted lowland water saving practice and IR64 is a popular, widely grown, high yielding mega rice variety for lowland irrigated system with the similar growth duration as that of genotypes under study, we made comparisons for root traits as well as yield with IR64. Like any other upland genotype, Kali Aus and Aus 276 have well developed root system and comparison of root traits of lowland adapted genotype with upland adapted genotypes would not have been proper. At flowering stage, number of nodal root below 20 cm and deep root length appears to have an effect on yield. The percent increase in nodal root number and root dry weight from 15 to 22 DAT and thereafter 30 DAT to 50% flowering (Table 8) has major role to play in water-nutrient uptake under AWD and decreasing grain yield reduction. The study highlights the significance of percent increase in root traits after initiation of the AWD cycle till 50% flowering, grouping of root with grain yield and the responsiveness of genotype possessing such traits to provide high and stable yield across different ecosystems. These novel findings shall encourage plant breeders for the application of the identified traits in the development of better rice cultivars for AWD condition. The soil aerating practice (Thakur et al., 2011) and high root dry weight (Pascual and Wang, 2017) implies more uptake of water and nutrients (Ndiiri et al., 2012) from the soil through to the root system (Dobermann and Fairhurst, 2000; Kirk, 2004) and better remobilization of carbohydrates from the source (stems) to the sink (grain) characterize the key method of enhancing grain filling under AWD conditions (Yang and Zhang, 2010).

## Conclusions

AWD system of rice cultivation has huge potential to save water and increase crop productivity but has not become popular because currently available rice varieties showed yield reduction under AWD. The study identified the contribution of nodal roots and root dry weight at 10–20 cm depth on 22–30 DAT in preventing yield reduction under AWD compared to flooded irrigation system. The findings shall encourage plant breeders to apply selection for these two traits in the development of rice cultivars for AWD condition. Identification of novel rice genotypes with high, stable yield under AWD across ecosystems shall help disseminate and popularize AWD system among farmers and achieve on average 20% water saving in rice cultivation without any yield reduction.

## Availability of data and materials

The data sets supporting the results of this article are included within the article.

## Author contributions

NS was involved in conducting the experiment, analysis, data interpretation, and drafting of the manuscript; SS, VL, BCo, PP, PM, and MC were involved in conducting the experiment at IRRI; RY and BCh were involved in conducting the experiment at NRRP, Hardinath (Nepal); HP was involved in conducting the experiment at RARS, Tarahara (Nepal); KI was involved in conducting the experiment at BRRI, (Bangladesh); KB and MR were involved in conducting the experiment at RRS, Kaul (India); CV was involved in conducting the experiment at Hyderabad (India); TT and VT were involved in conducting the experiment at Phnom Penh (Cambodia); KR was involved in data analysis; AK conceived the study and was involved in critical revision of the manuscript and final approval of the version to be published.

### Conflict of interest statement

The authors declare that the research was conducted in the absence of any commercial or financial relationships that could be construed as a potential conflict of interest.

## Supplementary material

Supplementary Figure 1 AWD pipes and system for scheduling irrigation: **(A)** when water table level is at the level of surface; **(B)** when water table level is >10 cm; **(C)** when water table level is >15 cm.

Supplementary Figure 2 Water table level in 2016WS averaged from three AWD pipe installed in zig-zag manner in field.
