# Supplementary figures and images for: Root Traits Enhancing Rice Grain Yield under Alternate Wetting and Drying Condition
Source: Front Plant Sci. 2017 Oct 31;8:1879. doi: 10.3389/fpls.2017.01879 (PMC5671499; doi:10.3389/fpls.2017.01879)

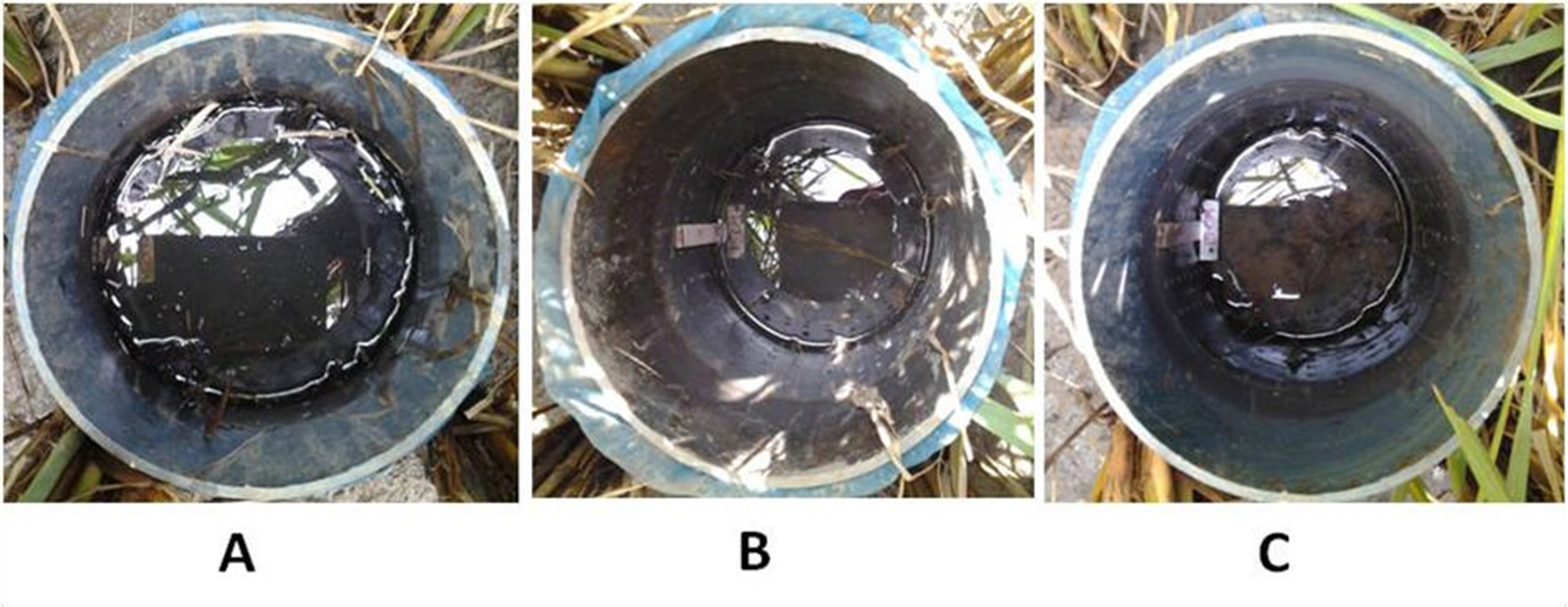

Supplement: Supplementary Figure 1 — AWD pipes and system for scheduling irrigation: (A) when water table level is at the level of surface; (B) when water table level is >10 cm; (C) when water table level is >15 cm. [file Image1.TIF]

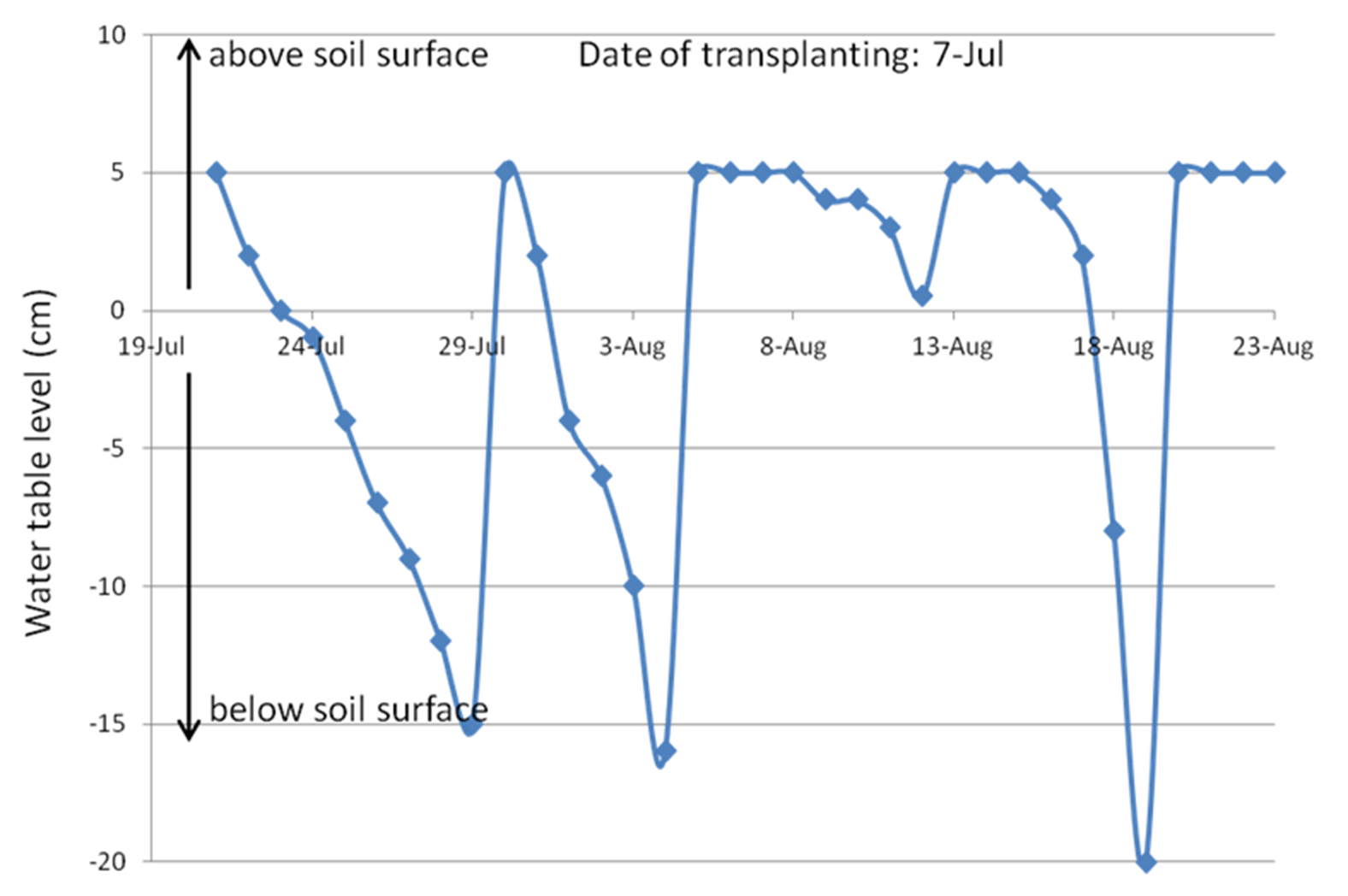

Supplement: Supplementary Figure 2 — Water table level in 2016WS averaged from three AWD pipe installed in zig-zag manner in field. [file Image2.TIF]
