# Supplementary material for: Root Traits Enhancing Rice Grain Yield under Alternate Wetting and Drying Condition
Source: Front Plant Sci. 2017 Oct 31;8:1879. doi: 10.3389/fpls.2017.01879 (PMC5671499; doi:10.3389/fpls.2017.01879)
Supplement: Supplementary file 3 [file Table1.DOCX]

**Supplementary Material**

**Rice root traits and varieties for stable yield under alternate wetting and drying condition in light of climate change in Asia**

Nitika Sandhu^1^, Sushil Raj Subedi^1^, Ram Baran Yadaw^2^, Bedanand Chaudhary^2^, Hari Prasai^3^, Khandakar Iftekharuddaula^4^, Tho Thanak^5^, Vathany Thun^5^, Khushi Ram Battan^6^, Mangat Ram^6^, Challa Venkateshwarlu^7^, Vitaliano Lopena^1^, Paquito Pablico^1^, Paul Cornelio Maturan^1^, Ma. Teresa Sta. Cruz^1^, K. Anitha Raman^1^, Bertrand Collard^1^, Arvind Kumar^1^*

^1^International Rice Research Institute, Los Baños, Laguna 4031, Philippines

^2^National Rice Research Program, Hardinath, Nepal

^3^Regional Agriculture Research Station, Tarahara, Nepal

^4^[Bangladesh Rice Research Institute](http://www.brri.gov.bd/), Gazipur, Bangladesh

^5^Cambodian Agricultural Research and Development Institute, Phnom Penh, Cambodia

^6^Rice Research Station, Kaul, India

^7^South Asia Breeding Hub, International Rice Research Institute, ICRISAT, Hyderabad, India

*** Correspondence:**

Arvind Kumar

a.kumar@irri.org

**Supplementary Table S1** **Details on year crossed, number of final families selected for screening and parentage of the advanced breeding lines used in the present study.**

| **Sr. No.** | **Year crossed** | **No of final cross selected for screening under AWD and NS** | **Parentage** |
| --- | --- | --- | --- |
| 1 | 2005DS | 2 | IR 71700-247-1-1-2/Samba Mahsuri, IR 77429-38-69-B-6-1-1/NSIC RC 138//IR 55423-01 (NSICRc 9) |
| 2 | 2006DS | 1 | IR 71606-1-1-4-2-3-1-2 (NSIC 110/IR 73459-120-2-2-3 |
| 3 | 2006WS | 2 | IR01W106/IR 71676-90-2-2, IRRI 143/IR 73718-23-2-1-3//IR00A110 |
| 4 | 2007WS | 9 | BR 29/Janaki, IR04A427/BR 29, IR04A427/IR 65450-173-2-1-1-3-3, IR04A427/Matang 1, IR04A427/IR 72875-94-3-3-2, IR04A427/IR 73006-12-3-3-2, IR04A427/PSBRc 52, IR05N173/BR 29, IR 80410-B-197-4/IRRI 149//NSICRc158 |
| 5 | 2008DS | 2 | IRRI 123/IR05N372, IR05N173/IRRI 143//IR 67966-44-2-3-2 |
| 6 | 2008WS | 2 | Matang 9/IR08L118, IR01N149/IR 64680-81-2-2-1-3//FEDEARROZ 50 |
| 7 | 2009DS | 4 | IR04A212/IR08L119, IR08L183/MTU 1010, IR02N211/TEQING, IR09N530/IR04A428 |
| 8 | 2009WS | 4 | IR08L119/IR 64, IR08L118/MTU 1010, Thadokkham 1/IR 77298-14-1-2-10, Thadokkham 1/IR08L119 |
| 9 | 2010DS | 10 | IR 71700-247-1-1-2/IR 77298-5-6-18,IR 71700-247-1-1-2/IR09L224, IR 72022-46-2-3-3-2/IR 77298-5-6-18, IR 72022-46-2-3-3-2/IR09L224, IR 78985-B-6-B-B-B/IR 81063-B-94-U 3-1, IR 81039-B-173-U 3-3/IR 81063-B-94-U 3-1,IR10N102/IR 86931-B-400, IR09L324/IR 78875-176-B-2, SANHUANGZHAN NO 2/IR 4630-22-2-5-1-3//FEDEARROZ 50/NSIC RC 158///IRRI 123/IR 45427-2B-2-2B-1-1//IR 77298-14-1-2-10/IR07F287, IR10N103/IRRI 150 |
| 10 | 2010WS | 16 | IR09L242/IR09L343, IR09L224/IR09L343, IR10L179/IR10L151, IR10L149/IR10L152, IR09L179/IR10L105, IR10L128/IR05N173, IR10L149/IR08N158, IR10L128/IR09N520, IR09N495/IR10L185, IR09N520/IR10L185, IRRI 168/IR10L105, IR09N495/IR10L105, IR08N158/IR10L105, IR 81896-B-B-236/IR 64,IR 77298-14-1-2-10/Q 74//IR 84984-83-15-18-B-B/Q 74, IR04A428/BR 29 |
| 11 | 2011DS | 15 | IR09L337/IR09L154, IR09L272/IR10L149, IR09L272/IR10L128, IR09L272/IR10L137, IR09L272/IR09L337, IR09L272/IR10L165, IR09L272/IR08L181, IR09L317/IR10L149, IR10L146/IR10L149, IR10L149/IR10L137, IR10L149/IR10L165, IR05N173/IR09L272, IR08N158/IR09L272, IR05N173/IR10L149, IR 97982:111/MRQ 74 |

*DS: dry season, WS: wet season*
