# Supplementary material for: Root Traits Enhancing Rice Grain Yield under Alternate Wetting and Drying Condition
Source: Front Plant Sci. 2017 Oct 31;8:1879. doi: 10.3389/fpls.2017.01879 (PMC5671499; doi:10.3389/fpls.2017.01879)
Supplement: Supplementary file 4 [file Table2.DOCX]

**Supplementary Material**

**Rice root traits and varieties for stable yield under alternate wetting and drying condition in light of climate change in Asia**

Nitika Sandhu^1^, Sushil Raj Subedi^1^, Ram Baran Yadaw^2^, Bedanand Chaudhary^2^, Hari Prasai^3^, Khandakar Iftekharuddaula^4^, Tho Thanak^5^, Vathany Thun^5^, Khushi Ram Battan^6^, Mangat Ram^6^, Challa Venkateshwarlu^7^, Vitaliano Lopena^1^, Paquito Pablico^1^, Paul Cornelio Maturan^1^, Ma. Teresa Sta. Cruz^1^, K. Anitha Raman^1^, Bertrand Collard^1^, Arvind Kumar^1^*

^1^International Rice Research Institute, Los Baños, Laguna 4031, Philippines

^2^National Rice Research Program, Hardinath, Nepal

^3^Regional Agriculture Research Station, Tarahara, Nepal

^4^[Bangladesh Rice Research Institute](http://www.brri.gov.bd/), Gazipur, Bangladesh

^5^Cambodian Agricultural Research and Development Institute, Phnom Penh, Cambodia

^6^Rice Research Station, Kaul, India

^7^South Asia Breeding Hub, International Rice Research Institute, ICRISAT, Hyderabad, India

*** Correspondence:**

Arvind Kumar

a.kumar@irri.org

**Supplementary Table S2.** **Grain yield stability of genotypes across ecosystems based on the best fitting (Finlay-Wilkinson regression model) variance–covariance structure according to the Akaike Information Criterion (AIC)**

| Designation | Estimate | Standard Error | Z Value | Pr Z | Rescaled beta | Mean grain yield (t/ha) across locations |
| --- | --- | --- | --- | --- | --- | --- |
| IR10N271 | 0.3675 | 0.2892 | 1.27 | 0.2039 | 0.23 | 4.3 |
| IR13A505 | 0.5472 | 0.3023 | 1.81 | 0.0703 | 0.35 | 3.6 |
| IR10F559 | 0.598 | 0.308 | 1.94 | 0.0522 | 0.38 | 4.1 |
| IR13A528 | 0.7306 | 0.3199 | 2.28 | 0.0224 | 0.46 | 5.2 |
| IR11N205 | 0.8371 | 0.3295 | 2.54 | 0.0111 | 0.53 | 3.6 |
| IR04A428 | 0.8966 | 0.336 | 2.67 | 0.0076 | 0.57 | 3.9 |
| IR14L148 | 0.9096 | 0.2489 | 3.65 | 0.0003 | 0.58 | 5.1 |
| IR14L269 | 1.0131 | 0.2177 | 4.65 | <.0001 | 0.64 | 5.4 |
| MTU1010 | 1.0256 | 0.3163 | 3.24 | 0.0012 | 0.65 | 5.1 |
| IR11A297 | 1.0594 | 0.3556 | 2.98 | 0.0029 | 0.67 | 4.6 |
| IR11A306 | 1.1051 | 0.328 | 3.37 | 0.0008 | 0.70 | 5.0 |
| IR14L248 | 1.1135 | 0.2956 | 3.77 | 0.0002 | 0.71 | 4.3 |
| IR10A231 | 1.1206 | 0.2387 | 4.69 | <.0001 | 0.71 | 5.1 |
| TDK11 | 1.1588 | 0.3484 | 3.33 | 0.0009 | 0.73 | 4.7 |
| IR14L111 | 1.1712 | 0.2487 | 4.71 | <.0001 | 0.74 | 5.6 |
| IR14L161 | 1.1754 | 0.2809 | 4.18 | <.0001 | 0.75 | 5.2 |
| IR14L255 | 1.1849 | 0.3073 | 3.86 | 0.0001 | 0.75 | 3.5 |
| IR14L245 | 1.1868 | 0.3073 | 3.86 | 0.0001 | 0.75 | 4.1 |
| IR14L138 | 1.1969 | 0.3091 | 3.87 | 0.0001 | 0.76 | 4.4 |
| IR11A292 | 1.2327 | 0.3202 | 3.85 | 0.0001 | 0.78 | 5.3 |
| IR14L257 | 1.294 | 0.3026 | 4.28 | <.0001 | 0.82 | 5.1 |
| IR14L156 | 1.2954 | 0.2735 | 4.74 | <.0001 | 0.82 | 5.1 |
| IR14L145 | 1.2998 | 0.2745 | 4.74 | <.0001 | 0.82 | 4.9 |
| IR14L247 | 1.3038 | 0.3269 | 3.99 | <.0001 | 0.83 | 4.3 |
| IR14L146 | 1.3476 | 0.284 | 4.75 | <.0001 | 0.85 | 5.6 |
| IR 09L226 | 1.3548 | 0.3224 | 4.2 | <.0001 | 0.86 | 4.7 |
| IR14L133 | 1.3626 | 0.2841 | 4.8 | <.0001 | 0.86 | 4.8 |
| IR11N202 | 1.3672 | 0.3994 | 3.42 | 0.0006 | 0.87 | 4.4 |
| IR14L272 | 1.3873 | 0.319 | 4.35 | <.0001 | 0.88 | 4.5 |
| IR13N104 | 1.3888 | 0.4016 | 3.46 | 0.0005 | 0.88 | 4.0 |
| IR14L110 | 1.3945 | 0.3205 | 4.35 | <.0001 | 0.88 | 5.1 |
| IR14L274 | 1.3962 | 0.2936 | 4.76 | <.0001 | 0.89 | 5.3 |
| IR14L244 | 1.4167 | 0.3258 | 4.35 | <.0001 | 0.90 | 4.8 |
| IR13L313 | 1.4195 | 0.3132 | 4.53 | <.0001 | 0.90 | 3.9 |
| IR13L337 | 1.4265 | 0.2995 | 4.76 | <.0001 | 0.90 | 4.9 |
| IR06N155 | 1.4269 | 0.4075 | 3.5 | 0.0005 | 0.90 | 4.2 |
| IR14L266 | 1.4407 | 0.2992 | 4.82 | <.0001 | 0.91 | 5.3 |
| IR14L108 | 1.4435 | 0.3028 | 4.77 | <.0001 | 0.92 | 4.9 |
| IR14L259 | 1.4499 | 0.3322 | 4.36 | <.0001 | 0.92 | 5.5 |
| IR09A235 | 1.483 | 0.4145 | 3.58 | 0.0003 | 0.94 | 4.3 |
| IR14L155 | 1.493 | 0.3127 | 4.77 | <.0001 | 0.95 | 5.0 |
| IR11A303 | 1.5056 | 0.3157 | 4.77 | <.0001 | 0.95 | 5.0 |
| IR12L321 | 1.5058 | 0.3157 | 4.77 | <.0001 | 0.95 | 5.0 |
| IRRI 123 | 1.5128 | 0.3172 | 4.77 | <.0001 | 0.96 | 5.3 |
| IR14L158 | 1.5389 | 0.322 | 4.78 | <.0001 | 0.98 | 4.9 |
| IR14L273 | 1.5486 | 0.3486 | 4.44 | <.0001 | 0.98 | 5.3 |
| IR11A282 | 1.5562 | 0.3994 | 3.9 | <.0001 | 0.99 | 4.9 |
| IR14L253 | 1.5598 | 0.3263 | 4.78 | <.0001 | 0.99 | 5.5 |
| IR11N313 | 1.5662 | 0.328 | 4.78 | <.0001 | 0.99 | 5.6 |
| IR14L249 | 1.5759 | 0.3298 | 4.78 | <.0001 | 1.00 | 5.4 |
| IR14L157 | 1.5789 | 0.3532 | 4.47 | <.0001 | 1.00 | 4.7 |
| IR14L135 | 1.5851 | 0.3545 | 4.47 | <.0001 | 1.00 | 4.4 |
| IR12N135 | 1.6164 | 0.4374 | 3.7 | 0.0002 | 1.02 | 4.6 |
| IR14L545 | 1.6394 | 0.3631 | 4.52 | <.0001 | 1.04 | 5.3 |
| IR11A334 | 1.6405 | 0.4426 | 3.71 | 0.0002 | 1.04 | 5.2 |
| IR14L252 | 1.6423 | 0.3668 | 4.48 | <.0001 | 1.04 | 4.8 |
| IR14L251 | 1.6804 | 0.3529 | 4.78 | <.0001 | 1.07 | 5.2 |
| IR14L360 | 1.7098 | 0.7805 | 2.19 | 0.0285 | 1.08 | 5.6 |
| IR14L143 | 1.7127 | 0.3976 | 4.31 | <.0001 | 1.09 | 3.9 |
| IR14L258 | 1.7333 | 0.3616 | 4.79 | <.0001 | 1.10 | 5.1 |
| IR14L297 | 1.7477 | 0.7921 | 2.21 | 0.0273 | 1.11 | 4.9 |
| IR14L420 | 1.7488 | 0.7722 | 2.26 | 0.0235 | 1.11 | 5.5 |
| IR14L530 | 1.7491 | 0.7812 | 2.24 | 0.0252 | 1.11 | 4.9 |
| IR14L246 | 1.7767 | 0.3704 | 4.8 | <.0001 | 1.13 | 5.2 |
| IR11A192 | 1.7781 | 0.464 | 3.83 | 0.0001 | 1.13 | 4.3 |
| IR14L583 | 1.8933 | 0.8739 | 2.17 | 0.0303 | 1.20 | 4.7 |
| IR14L374 | 1.9518 | 0.8137 | 2.4 | 0.0165 | 1.24 | 5.4 |
| IR14L144 | 2.0562 | 0.4448 | 4.62 | <.0001 | 1.30 | 4.8 |
| IR05N412 | 2.0974 | 0.513 | 4.09 | <.0001 | 1.33 | 4.2 |
| IR06A145 | 2.1067 | 0.4946 | 4.26 | <.0001 | 1.34 | 5.2 |
| IR 64 | 2.1486 | 0.5971 | 3.6 | 0.0003 | 1.36 | 4.5 |
| IR14L586 | 2.179 | 0.6866 | 3.17 | 0.0015 | 1.38 | 5.4 |
| IR14L395 | 2.2884 | 0.856 | 2.67 | 0.0075 | 1.45 | 5.4 |
| IR14L345 | 2.4199 | 0.89 | 2.72 | 0.0065 | 1.53 | 4.7 |
| IR14L613 | 2.4377 | 0.863 | 2.82 | 0.0047 | 1.55 | 4.8 |
| IR14L601 | 2.5102 | 0.8862 | 2.83 | 0.0046 | 1.59 | 5.0 |
| IR12N234 | 2.6073 | 0.5889 | 4.43 | <.0001 | 1.65 | 5.1 |
| IR14L440 | 2.625 | 0.9993 | 2.63 | 0.0086 | 1.66 | 4.9 |
| IR10N136 | 2.7338 | 0.6121 | 4.47 | <.0001 | 1.73 | 5.0 |
| IR14L362 | 2.7583 | 0.9448 | 2.92 | 0.0035 | 1.75 | 5.1 |
| IR14L604 | 3.174 | 0.985 | 3.22 | 0.0013 | 2.01 | 4.8 |
| IR14L331 | 3.3335 | 1.3482 | 2.47 | 0.0134 | 2.11 | 5.0 |
